# The Use of Amnion-Derived Cellular Cytokine Solution to Improve Healing in Acute and Chronic Wound Models

**Published:** 2008-04-11

**Authors:** Michael G Franz, Wyatt G Payne, Liyu Xing, D. K Naidu, R. E Salas, Vivienne S Marshall, C. J Trumpower, Charlotte A Smith, David L Steed, M. C Robson

**Affiliations:** University of Michigan, Ann Arbor, MI; Institute for Tissue Regeneration, Repair, Rehabilitation, Department of Veterans Affairs Medical Center, Bay Pines, FL; Stemnion, Inc.; University of Pittsburgh, Pittsburgh, PA

## Abstract

**Objective:** Growth factors demonstrate mixed results improving wound healing. Amnion-derived multipotent cells release physiologic levels of growth factors and tissue inhibitors of metalloproteinases. This solution was tested in models of acute and chronic wound healing. **Methods:** *Acute model: Sprague-Dawley* rats underwent laparotomy incisions. The midline fascia was primed with phosphate-buffered saline, unconditioned media, or amnion-derived cellular cytokine suspension prior to incision. Breaking strength of laparotomy wounds was tested with an Instron tensiometer. Incisional hernia formation was measured after 28 days. *Chronic model:* Chronic, infected granulating wounds were produced in rats by excising full thickness burn eschars inoculated with *Escherica coli.* Granulating wounds were treated with unconditioned media or amnion-derived cellular cytokine suspension. Treatments were applied either on day 0 and day 7 or day 0 and then every other day. Wounds were traced every 72 hours and biopsied for quantitative bacteriology. **Results:** *Acute model:* Priming with amnion-derived cellular cytokine suspension increased the breaking strength of laparotomy incisions in comparison with phosphate-buffered saline or unconditioned media (*P* < .05). Acute wound failure and incisional hernia formation was 100% in the phosphate-buffered saline and unconditioned media groups and 18% in the amnion-derived cellular cytokine suspension–treated group (*P* <.05). *Chronic model:* The rate of wound closure was accelerated in amnion-derived cellular cytokine suspension–treated chronic wounds (*P* < .05). Multidosing improved the effect. **Conclusions:** A physiologic solution of cytokines and tissue inhibitors of metalloproteinases improves healing in models of acute and chronic wounds. Such a cocktail can be produced from amnion-derived multipotent progenitor cells.

Stem cells or stem cell–like multipotent cells have great potential in the wound healing/tissue repair arena. They have the ability to differentiate into the various cell types of the repair process and to secrete the humoral messengers necessary to mediate the cellular processes.^1^–^3^ Adult stem-like cells derived from amnion have been shown to secrete many cytokines and growth factors.^4^–^6^

Amnion-derived multipotent progenitor (AMP) cells were recently reported to increase the rate of gain of incisional breaking strength and decrease the incidence and severity of acute wound failure.^7^ It was postulated that one of the possible mechanisms for the improvement of acute wound healing in these experiments was that the AMP cells could be making the necessary cytokine cocktail at the proper time and dose to affect a trajectory shift.^7^ The secreted cytokines from AMP cells, amnion-derived cellular cytokine solution (ACCS), have been studied using qualitative antibody arrays, enzyme-linked immunosorbent assays, multiplex, and mass spectroscopy. The solution was found to contain a combination of cytokines and growth factors at physiological levels.^8^ There was a good correlation between the available levels of cytokines reported as measured in humans in normal and diseased states and the level of cytokines found to be secreted by the AMP cells. In most cases, cytokine and growth factor concentrations measured in ACCS were near the lower end of reported physiological ranges. The exception was for tissue inhibitor of metalloproteinase-1 (TIMP-1) and tissue inhibitor of metalloproteinase-2 (TIMP-2), which were detected in ACCS at the upper limit of known physiological levels.

The purpose of the present study was to evaluate the effects of local application of ACCS in both acute and chronic wound healing models.

## MATERIALS AND METHODS

AMP cells were grown to confluency, and using proprietary techniques, the supernatant was harvested.^8^ This secreted product, labeled ACCS, was then used in both the acute wound model and the chronic wound model. Detailed analysis of the secreted growth factor and cytokine profile, including, but not limited to, platelet-derived growth factor-BB, vascular endothelial growth factor, angiogenin, TIMP-1, and TIMP-2 were previously reported.^8^

### Acute wound model

#### Animal model

The rat models of laparotomy wound healing (W) and incisional hernias (H) were previously reported.^9^,^10^ Sprague-Dawley rats (Harlan Inc, Indianapolis, Ind) weighing 450 to 500 g were acclimated and housed under standard conditions. Animals were allowed ad libitum intake of standard rat chow and water throughout the study. All animal care and operative procedures were performed in accordance with the United States Public Health Service *Guide for the Care of Laboratory Animals*^11^ and were approved by the University Committee on Use and Care of Animals of the University of Michigan.

#### Laparotomy wound healing model

Briefly, a 6 × 3 cm ventral full-thickness skin flap is raised through the avascular prefascial plane, and a 5-cm full-thickness laparotomy incision is made through the linea alba at the musculo-tendinous layer of the abdominal wall. The laparotomy wound is repaired with a running 4-0 polypropylene suture, using 0.3-cm suture bites and 0.5-cm progress between stitches. The suture is tied to itself at the end of the wound. The skin flap is sutured in place with three 4-0 polypropylene stitches and steel wound clips. After 30 minutes of recovery under a heat lamp, the rats are returned to fresh individual cages.

Three laparotomy wound groups were studied: (1) a phosphate-buffered saline–treated wound (PBS-W); (2) an unconditioned media–treated wound (UCM-W); and (3) an ACCS-treated wound (ACCS-W).^8^ In all 3 groups, the midline of the abdominal wall (linea alba) was injected for a length of 5 cm with 200 μL of PBS, UCM, or ACCS. This surgical site priming was achieved with a 22-gauge hypodermic needle (0.7 × 38 mm) into the linea alba, as previously reported.^7^ Soft tissue distribution of PBS, UCM, or ACCS suspension was achieved. After 5 minutes, the laparotomy incision was made and repaired as described above.

On postoperative day (POD) 7, POD 14, and POD 28, the rats were killed. Isolated abdominal wall muscle and tendon strips and fresh biopsies of the abdominal wall to laparotomy wound-healing interface were collected for mechanical and histological testing.

#### Laparotomy wound breaking strength and mechanical properties

Mechanical testing was performed on abdominal wall strips collected from the laparotomy wound healing model as previously reported.^10^,^12^,^13^ All sutures were removed. Abdominal wall strips were cut perpendicular to the wound-healing interface. A cutting template was used to mark the abdominal wall to minimize size variability between specimens. Strips were cut 10 mm in width and 60 to 80 mm in length. Two strips were collected from each rat and testing was performed within 6 hours of necropsy. The fascial tissue strip thickness at the wound and the length between grips were measured with Digimatic calipers (Mitutoyo America Corp, Chicago, Ill). Stretch loading facilitated mechanical characterization of the wound-healing interface. Force extension curves were generated for each fascial strip with the use of an Instron Tensiometer (model 5542; Instron Corporation, Canton, Mass) equipped with a 50-N static load cell set at a crosshead speed of 10 mm/min. The fascial strips were mounted into the load frame with the use of pneumatic grips, preloaded to 0.1 N with the gauge length measured between the grips that was around 10 mm. The load frame applied testing loads to the fascial strips until mechanical tissue disruption occurred. The anatomic location of the break was noted for each specimen. Force and tissue deformation data were simultaneously recorded and captured on a computer connected to the load frame via a digital interface card. Data analysis was performed with the use of the Merlin materials testing software package (Instron Corporation) from which breaking strength, the maximum load force (F_max_ at mechanical failure (in newton) and tensile strength, the maximum stress developed in the specimen per unit area, calculated as F_max_/cross-sectional area (/newton per square millimeter). Failure of the specimen was defined at the yield point rather than at the point of ultimate tissue disruption because the yield point indicates the region where the tissues are irreversibly deformed and mechanical defects first occur (ie, beyond the elastic limit of the tissues).

#### Incisional hernia model

Mechanically, failing laparotomy incisions form incisional hernias. Clinically, this manifests as defects in the musculo-tendinous-peritoneal layer of the abdominal wall. The serious clinical consequences of this are acute abdominal wall dehiscence and evisceration, the incarceration and obstruction of peritoneal viscera, loss of the ability of the abdominal wall to maintain torso posture, and chronic pain.

The incisional hernia models were used as previously reported.^9^,^10^ Briefly, a 6 × 3 cm ventral full-thickness skin flap was raised through the avascular prefascial plane. In each experimental group, 200 μL of PBS, UCM, or ACCS was injected along the midline for 5 cm prior to laparotomy incision, infiltrating the linea alba. Next, a 5-cm full-thickness laparotomy incision was made through the linea alba. Two 5-0 fast absorbing plain catgut stitches were placed at the cranial end and mid-point of the laparotomy incision. The skin flap was returned and sutured in place with three 4-0 polypropylene sutures and steel wound clips. The 3 experimental groups were (1) phosphate-buffered saline–primed hernia (PBS-H); (2) UCM-primed hernia (UCM-H); and (3) ACCS-primed hernia (ACCS-H). On POD 28, the rats were killed and the musculo-tendinous layer of the abdominal wall was collected and examined for mechanical wound failure and incisional hernias.

#### Measurement of hernia size

For hernia size measurement, the hernia model rats were killed on POD 28. The skin was dissected free circumferentially, and 5 × 10 cm of the abdominal wall muscle was excised. The muscle was stretched out and pinned down on a dissecting board at the 4 corners with the peritoneal side up. A ruler was set alongside the wound as a reference for every sample. A standardized digital picture was then taken. Software Spot, Windows version 4.5 (Diagnostic Instruments, Inc., University of New South Wales, Sydney, Australia) was used to calculate the hernia size on digital pictures. Calibration was set up using the rule reference on each picture. A circle was drawn along the hernia ring to measure the hernia size as square centimeter, as previously reported.^7^.

#### Wound and tissue histology

Sagittal fascial laparotomy wound and/or hernia sections were then cut and immediately fixed in 10% neutral-buffered formalin in preparation for histologic analysis. Specimens were embedded in paraffin and sectioned and stained with hematoxylin and eosin or trichrome by a core research histology service or the Immunoperoxidase Laboratory in the University of Michigan Comprehensive Cancer Center.

## Chronic wound model

### Animal model

Chronic granulating wounds were prepared as previously described^14^–^16^ after approval of the protocol by the Institutional Animal Care and Use Committee of the Bay Pines Veterans Administration Medical Centre. This model has been histologically compared to the human chronic granulating wound.^17^ Forty male Sprague-Dawley rats weighing 300 to 350 g were acclimatized for 1 week in the animal facility prior to use. Under intraperitoneal pentobarbital (Nembutal) anesthesia, the rat dorsum was shaved and depilated. A full-thickness dorsal burn measuring 30 cm^2^ was created by immersion in actively boiling water.^14^,^17^ Seven milliliters of Ringer's lactate solution by subcutaneous injection was given to each rat to prevent dehydration. The wounds were treated in the group treatment scheme as follows:

- *Group I* received no other treatment and served as the uninfected control.
- *Group II* received no other treatment and served as the infected control.
- *Group III* was treated with ACCS on the day of escharectomy and 7 days later.
- *Group IV* was treated with ACCS on day of escharectomy and then every other day.
- *Group V* was treated with unconditioned media on day of escharectomy and 7 days later.

Rats in the contaminated groups (groups II–V) were seeded with 5 × 10^8^ colony forming units (CFUs)/mL *Escherichia coli* (ATCC 25922) after they had been allowed to cool for 15 minutes. Group I rats were not inoculated and served as the uninfected controls. Bacteria were obtained from fresh 18-hour broth cultures and inoculum size was confirmed by backplating. The animals were divided into 5 equal groups of 8 for different treatments after the day 5 escharectomies.

Animals were individually caged and given food and water ad libitum. Five days after burning, the eschars were excised from anesthetized animals, resulting in a chronic granulating wound. These wounds contained greater than 10^5^ bacteria/g of tissue (except for those in group I). Histologic characterization of this wound with comparison to a human granulating wound has previously been performed.^17^

Every 72 hours, animals were pretreated with buprinorphine (Buprenex) (0.1 mg/kg), anesthetized with halothane inhalation, and the outlines of the wounds were traced onto acetate sheets. Area calculations were performed using digital planimetry. Any dried exudate that formed was atraumatically removed prior to any wound tracings or biopsies. Care was taken to record only the advancing full-thickness margin rather than any advancing edge of epithelium. This avoided the small component of advancement provided by the smooth, pink, translucent, and hairless neoepithelium.^16^ Serial area measurements were plotted against time. Biopsies for quantitative and qualitative bacterial analyses according to the method of Heggers^18^ were performed on days 8, 17, and 28 postescharectomy to exclude superinfection and to confirm bacterial levels in infected animals.

## Statistics

### Acute model

Statistical analysis was performed using GraphPad Prism version 4.00 for Windows (GraphPad Software, San Diego, Calif, www.graphpad.com). *t* Test was used to compare the difference between the normal saline (NS) control group and the UCM- or ACCS-treated groups. This software was also used to create the incidence curves of incisional hernia at POD 28 for fractional hernia at any particular hernia or wound defect size and compare the curves among NS-H, UCM-H, and ACCS-H groups. Significant level was set at *P* < .05.

### Chronic model

For each animal's data, a Gompertz equation was fitted (typical *r*^2^ = 0.85).^19^ Using this curve, the wound half-life was estimated. Comparison between groups was performed using life table analysis and the Wilcoxon rank sum test. The statistical analyses were performed using *SAS/STAT User's Guide for Personal Computer*^20^ and *BMDP Statistical Software Manual*^21^ packages on a personal computer. From the best-fit curves for the individual wounds, the number of days required for 25%, 50%, and 75% healing of the original wounds were calculated.^19^

## RESULTS

### Acute wound model

#### ACCS accelerates wound healing in laparotomy incisions

**Figure 1:**
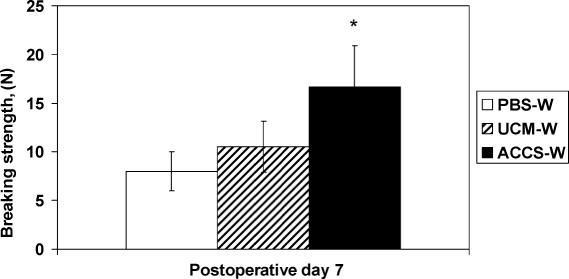
Tensiometric measurements of healing laparotomy incisions. Fascial wound breaking strength was measured with a calibrated Instron Tensiometer on postoperative day 7. Values are the mean ± SEM of 6 wound biopsies each from the phosphate-buffered saline–treated wound (PBS-W) and unconditioned media–treated wound (UCM-W) controls and the amnion-derived cellular cytokine suspension–treated wound (ACCS-W) groups. Analysis of variance was applied to measure the differences between the 3 groups. The asterisk indicates significance at *P* < .05.

**Figure 2:**
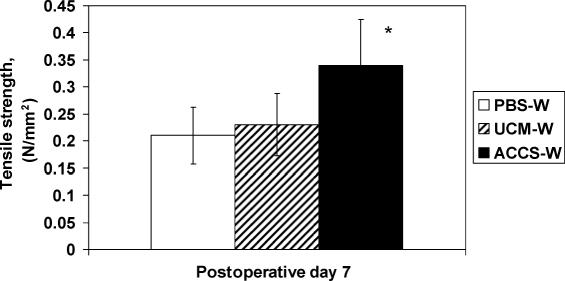
Tensiometric measurements of healing laparotomy incisions. Fascial wound tensile strength was measured with a calibrated Instron Tensiometer on postoperative day 7. Values are the mean ± SEM of 6 wound biopsies each from the phosphate-buffered saline–treated wound (PBS-W) and unconditioned media–treated wound (UCM-W) controls and the amnion-derived cellular cytokine suspension–treated wound (ACCS-W) groups. Analysis of variance was applied to measure the differences between the 3 groups. The asterisk indicates significance at *P* <.05.

**Figure 3:**
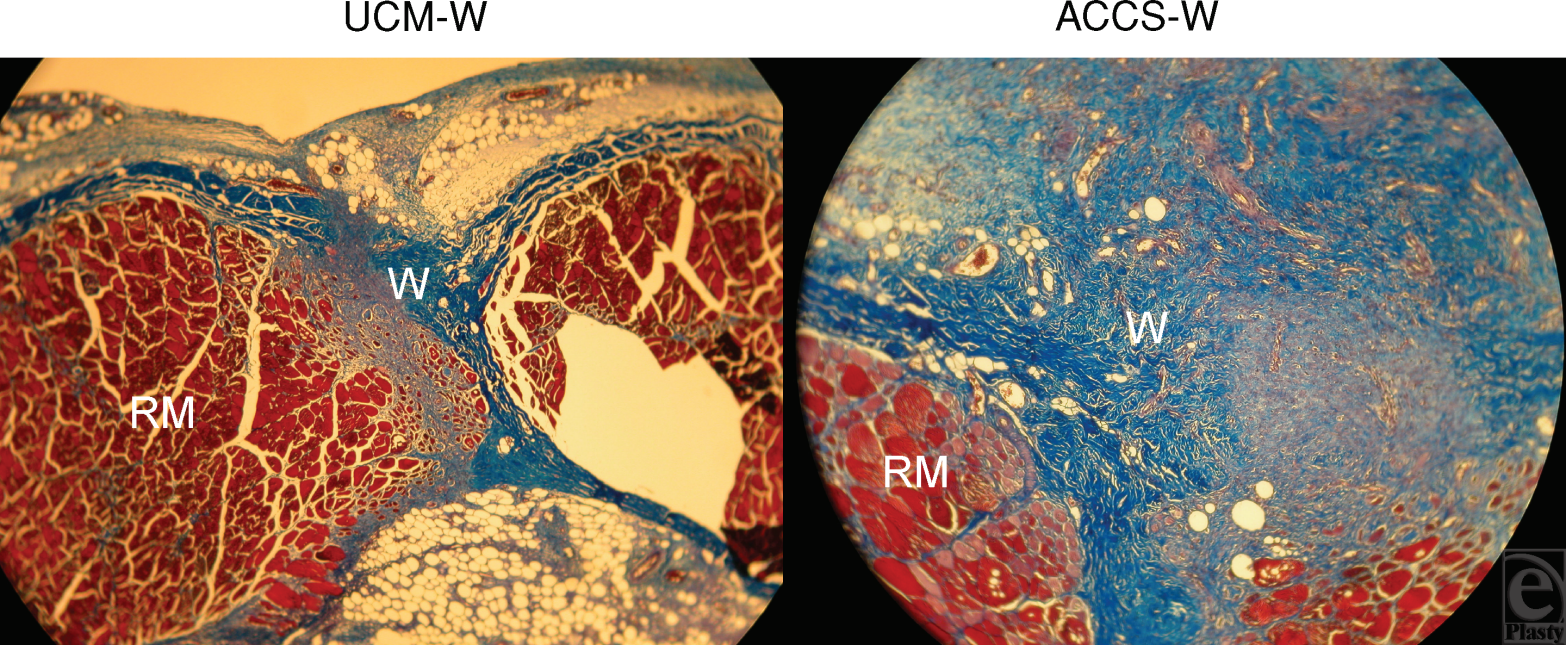
Morphology of fascial laparotomy wounds on postoperative day 7 (unconditioned media–treated wound [UCM-W] and amnion-derived cellular cytokine suspension–treated wound [ACCS-W] shown). Trichrome staining was performed for histology. ACCS-treated wounds developed more granulation tissue and organized fibro-proliferation than UCM-W groups. RM indicates rectus muscle and W, laparotomy wound.

The abdominal wall was treated with ACCS to measure its effect on laparotomy wound healing. Tensiometric analysis was performed on uniform abdominal wall strips with the line of tissue deformation directly perpendicular to the linea alba/incision line. Tensiometric measurements found significant differences in the mechanical properties of the wound among groups treated with the PBS-W, UCM-W, and ACCS-W. All wounds always mechanically disrupted at the fascial-fascial wound interface. Priming with ACCS accelerated the gain of breaking strength of laparotomy incisions in comparison with PBS or UCM (*P*<.05). As shown in Figure 1, wounds treated with 200 μL of ACCS per rat developed greater breaking strength by POD 7 in comparison to the PBS-W and UCM-W control groups. Tensile strength was also greater in the ACCS-W group on POD 7, suggesting a physiological effect on laparotomy wound scar quality (Fig 2). Wound samples harvested on POD 7 demonstrated more granulation tissue and organized fibro-proliferation with increased angiogenesis in the ACCS-W group than PBS-W or UCM-W groups (Fig 3).

#### ACCS reduces laparotomy dehiscence and hernia formation

**Figure 4:**
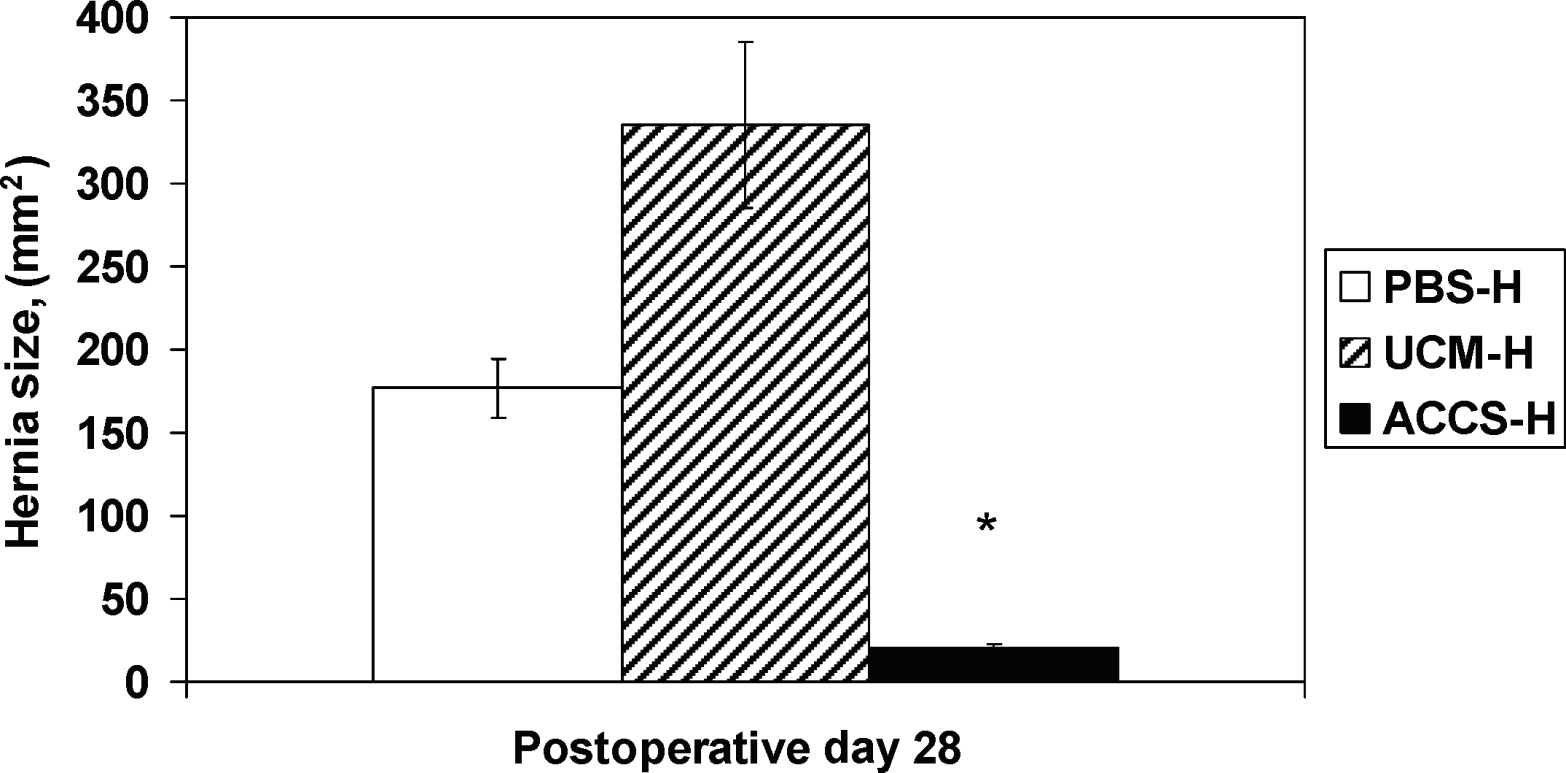
Measurement of hernia size. Digital pictures were taken with a ruler as reference for every wound sample. Software spot was employed to measure the calibrated wound defect and hernia sizes. A digital perimeter was drawn along the wound and hernia ring to calculate the area in square millimeters. Analysis of variance was applied to measure the differences between the 3 groups. The asterisk indicates significance at *P* <.05. PBS-H indicates phosphate-buffered saline–primed hernia; UCM-H, unconditioned media–primed hernia; and ACCS-H, amnion-derived cellular cytokine suspension–primed hernia.

**Figure 5:**
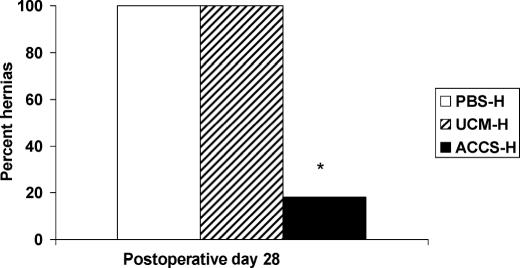
Amnion-derived cellular cytokine suspension treatment reduces the incidence and severity of laparotomy wound failure that progresses to an incisional hernia. The asterisk indicates significance at *P* <.05. PBS-H indicates phosphate-buffered saline–primed hernia; UCM-H, unconditioned media–primed hernia; and ACCS-H, amnion-derived cellular cytokine suspension–primed hernia.

On POD 28, the rate of acute wound failure leading to incisional hernia formation was 100% in the PBS-H and UCM-H groups and 18% in the ACCS-treated group (*P* < .05). In this model of intentional incisional hernia, the expected rate of incisional hernia formation is 80% to 100%.^9^,^10^,^12^ Clinically, incisional hernia of the laparotomy incision is a very common complication of the human abdominal wall. Clinical and experimental evidence support that the primary mechanism for incisional hernia formation is very early mechanical failure of the laparotomy wound.^9^,^13^ These studies have shown that there is a minimum mechanical wound failure size that predicts clinical incisional hernia formation with nearly 100% accuracy. In the human study, a fascial gap of 12 mm predicted incisional hernia formation 94% of the time, whereas mechanical gaps of less than 12 mm healed 99.2% of the time^22^ The conclusion is that small laparotomy defects do not form incisional hernias, but bigger ones do. In the current study, rats were killed on POD 28 and the abdominal wall was collected to measure directly laparotomy wound defect size and hernia formation. As shown in Figure 4, laparotomy wound defect size in the ACCS-H group was significantly smaller than those in the PBS-H or UCM-H groups (21 ± 4.3 mm^2^ versus 117 ± 11 mm^2^ versus 335 ± 23 mm^2^, respectively). Predictably, the incidence of incisional hernia formation was therefore also less in the ACCS-treated group (Fig 5). ACCS treatment therefore reduces the incidence of laparotomy wound failure that progresses to an incisional hernia.

#### Chronic wound model

**Figure 6:**
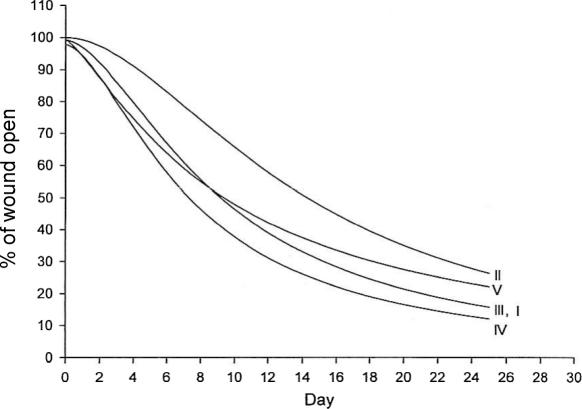
Chronic infected granulating wound. Amnion-derived cellular cytokine suspension (ACCS) treatment, both when administered on day 0 and day 7 or every other day, accelerated healing in comparison with the infected control (*P* <.05). More frequent applications of ACCS resulted in the most rapid decrease in wound size. Despite the presence of greater than 10^5^ CFUs/g of tissue in the amnion-derived cellular cytokine suspension–treated groups (III, IV), the wounds healed at least as rapidly as when no bacteria were present (group I). I indicates noninfected group; II, infected control; III, ACCS administered on day 0 and day 7; IV, ACCS administered on Monday, Wednesday, and Friday; and V, unconditioned medium.

The rate of wound closure was statistically accelerated in ACCS-treated chronic infected granulating wounds in comparison with the infected controls (*P* < .05). Applying ACCS more frequently further accelerated the healing (Fig 6). The bacterial bioburden in the wounds did not significantly change with ACCS treatment, remaining greater than greater than 10^5^ CFUs/g of tissue in groups II to V. This confirmed that the effect of ACCS was not because of antimicrobial action.

### DISCUSSION

Exogenous application of cytokines and growth factors has been reported for many animal models of both acute and chronic wounds.^23^ In almost all of these models, it has been suggested that wound healing would be enhanced by topical application of these peptides. Using the acute wound model employed in these studies, the effects of individual cytokines and their ability to decrease acute wound failure have been demonstrated.^12^,^24^,^25^ Although single growth factors have been successful in improving healing in this model, normal wound healing is accompanied by a combination of cytokines and they occur in a cascade.^8^,^26^,^27^ By using a combination of cytokines present in physiological levels, it appears that ACCS can improve wound healing. The concept behind platelet-derived wound healing factors reported by Knighton et al^28^ was to deliver a natural combination of cytokines. Unfortunately, the exact levels of cytokines present in platelet-derived wound healing factors were determined by its dilution, were not clearly delineated, nor were they compared to physiological levels of cytokines necessary for healing as has been done for ACCS.^8^ The purpose of the current study was to report an early clinical application using animal models. Because the endpoint is wound healing, the possible inhibitory effect of an isolated growth factor or cytokine was not measured. This is a theoretical possibility, but testing a panel of individual growth factors and cytokines, and then all possible combinations to tease out unique symbiotic or antagonistic effects would take a long time at great expense and may only yield uninterpretable data. Our rationale was that conditioned media (ACCS) containing physiologic combinations of naturally occurring growth factors derived from amnion cells would improve wound healing.

By shifting the wound healing trajectory to the left and gaining incisional strength more rapidly with ACCS treatment (Figs 1 and 2), the incidence of acute wound failure was decreased (Fig 5). Not only was the incidence decreased, the size of the resultant hernias when they did occur was smaller (Fig 4). Histologic sections suggest a more accelerated repair with ACCS treatment (Fig 3).

The chronic infected granulating wound model has also been used to evaluate individual cytokine growth factors including basic fibroblast growth factor,^14^,^15^ granulocyte-macrophage colony-stimulating factor,^29^ IL-4, 30 and keratinocyte growth factor-2.^31^ In each of these cases, large doses of the test proteins were required for an effect. It was postulated that this was because of destruction or neutralization of the cytokines by tissue and bacterial matrix metalloproteinases (MMPs).^32^ One reason that ACCS may have been effective in this model in physiological doses of cytokines is because of high levels of TIMP-1 and TIMP-2, known natural inhibitors to MMPs.^8^ These high levels of TIMPs improve the MMP/TIMP ratio, which has been reported to improve healing in chronic wounds.^33^ Tissue bacterial bioburden remained high (>10^5^ CFUs/g of tissue) in both treated and control wounds.

From the previous report on AMP cells in the acute wound model and these observations of ACCS treatment in both acute and chronic wounds, it appears that AMP cells and their secreted products in ACCS can form a novel platform technology for further investigation in the wound healing arena.

### ACKNOWLEDGMENTS

This study was supported by unrestricted grants from Stemnion, Inc. and Department of Defense (USAMRMC 0610000).
